# Peritoneal dissemination of breast cancer diagnosed by laparoscopy

**DOI:** 10.1007/s13691-020-00456-w

**Published:** 2020-11-14

**Authors:** Tsuyoshi Nakagawa, Goshi Oda, Akifumi Kikuchi, Toshifumi Saito, Tomoyuki Fujioka, Kazunori Kubota, Mio Mori, Iichiro Onishi, Hiroyuki Uetake

**Affiliations:** 1 Department of Breast Surgery, Tokyo Medical and Dental University, 1-5-45 Yushima Bunkyou-ku, Tokyo, 113-8519 Japan; 2 Department of Colorectal Surgery, Tokyo Medical and Dental University, Tokyo, Japan; 3 Department of Radiology, Tokyo Medical and Dental University, Tokyo, Japan; 4 Department of Pathology, Tokyo Medical and Dental University, Tokyo, Japan

**Keywords:** Breast cancer, Peritoneal dissemination, Laparoscopy

## Abstract

The accuracy of modern imaging techniques for the diagnosis of peritoneal carcinomatosis is poor. A breast cancer patient with a high serum CA15-3 level did not receive a definitive diagnosis of peritoneal dissemination by imaging examination and then underwent laparoscopy. Pathological examination showed peritoneal dissemination of breast cancer, but the biological markers were different from the primary lesion: ER(−), PgR(−), and Her2:3 +. T-DM1 therapy was very effective, and her systemic symptoms disappeared. Since biomarkers of metastatic lesions may sometimes change, laparoscopic biopsy is very important and useful.

## Introduction

Breast cancer is the most common cancer in women and can metastasize to many organ sites. In particular, peritoneal dissemination from breast cancer is uncommon and a life-threatening condition with a very high mortality rate. It has always been managed by systemic chemotherapy, exclusively with palliative intent. Moreover, the accuracy of modern imaging techniques for the diagnosis of peritoneal carcinomatosis is poor. The case of a patient with peritoneal dissemination from breast cancer diagnosed by laparoscopy is presented in which the biomarkers differed from those of the primary lesion on pathological examination, which could be very useful information for subsequent systemic treatment.

## Case report

A 68-year-old woman was diagnosed with left early breast cancer and underwent partial resection and sentinel lymph node biopsy. The pathological findings were as follows: T1cN0M0, invasive ductal carcinoma (papillotubular carcinoma), nuclear grade: 3, ER(+), PgR(−), and HER2:0. Adjuvant chemotherapy consisted of 4 cycles of EC (epirubicin and cyclophosphamide), and adjuvant hormonal therapy with anastrozole was scheduled for 10 years.

**Fig. 1:**
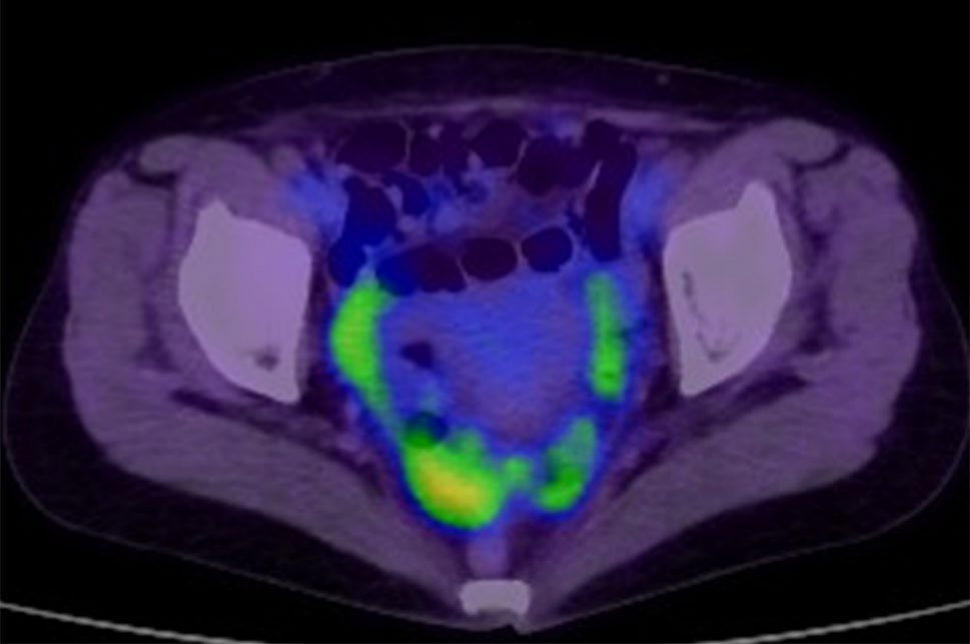
FDG-PET/CT shows slight FDG accumulation in the pelvic peritoneum, suggesting peritoneal dissemination of breast cancer

**Fig. 2:**
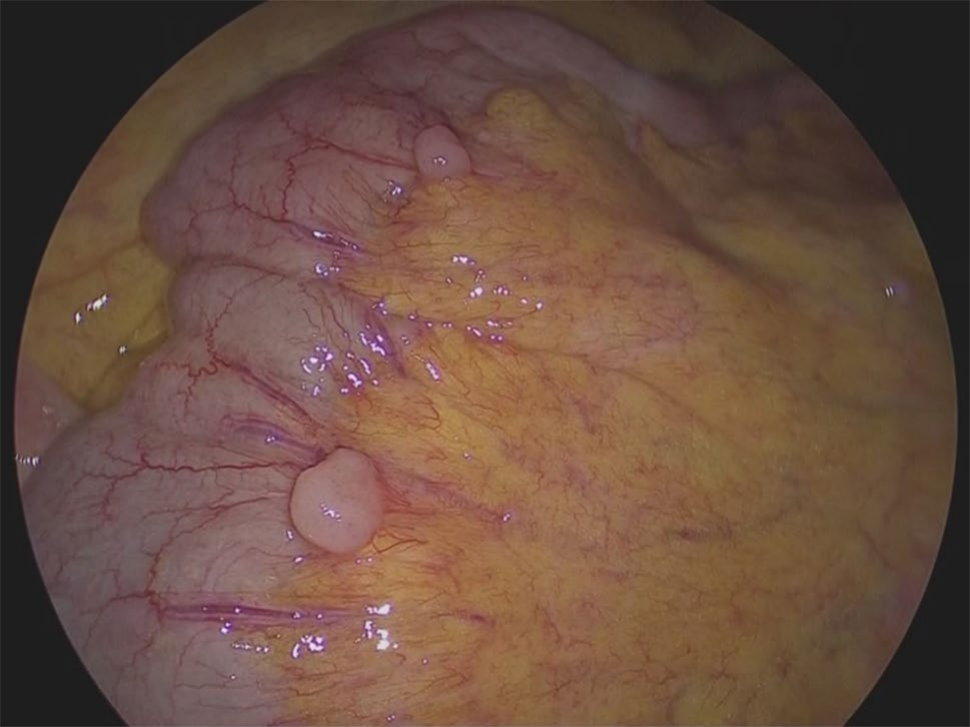
Peritoneal dissemination in the mesentery

**Fig. 3:**
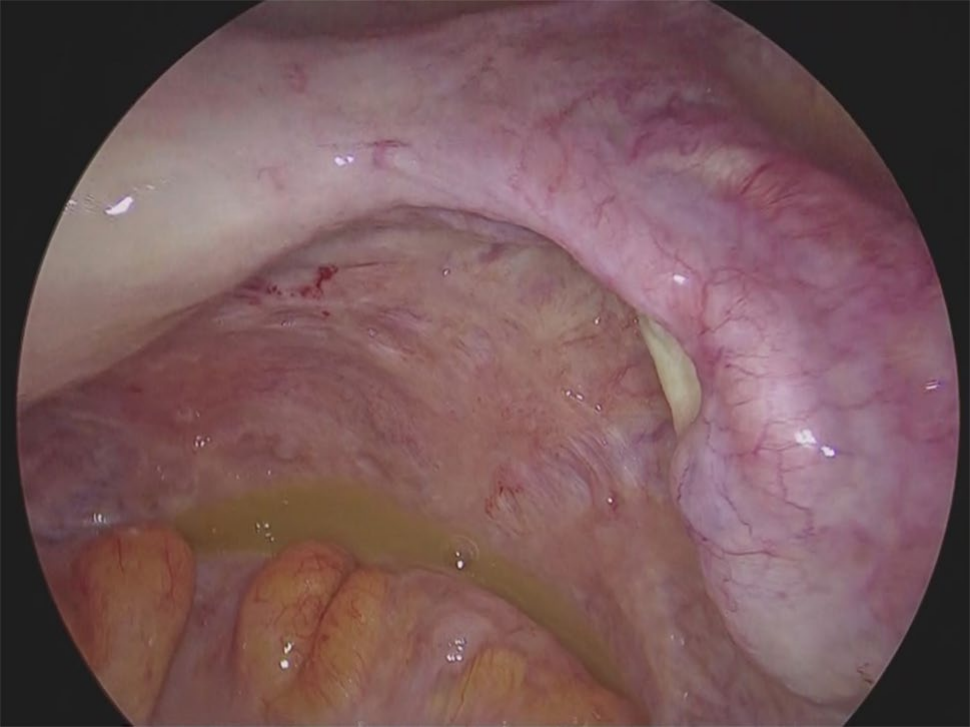
Ascites in the pelvis

**Fig. 4:**
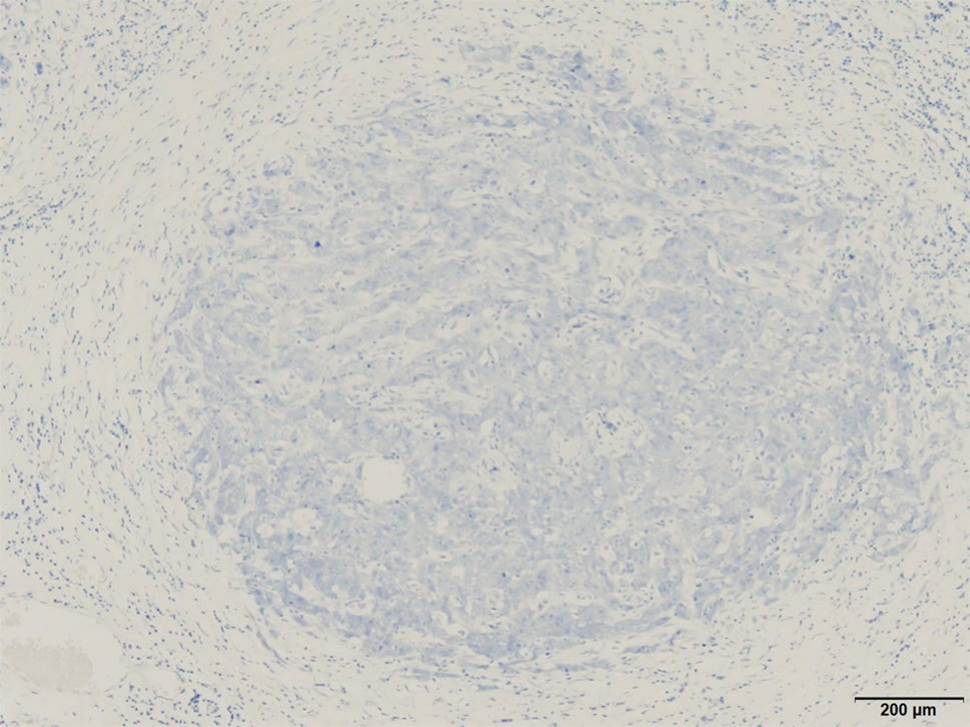
Negative HER2 expression in the primary lesion

**Fig. 5:**
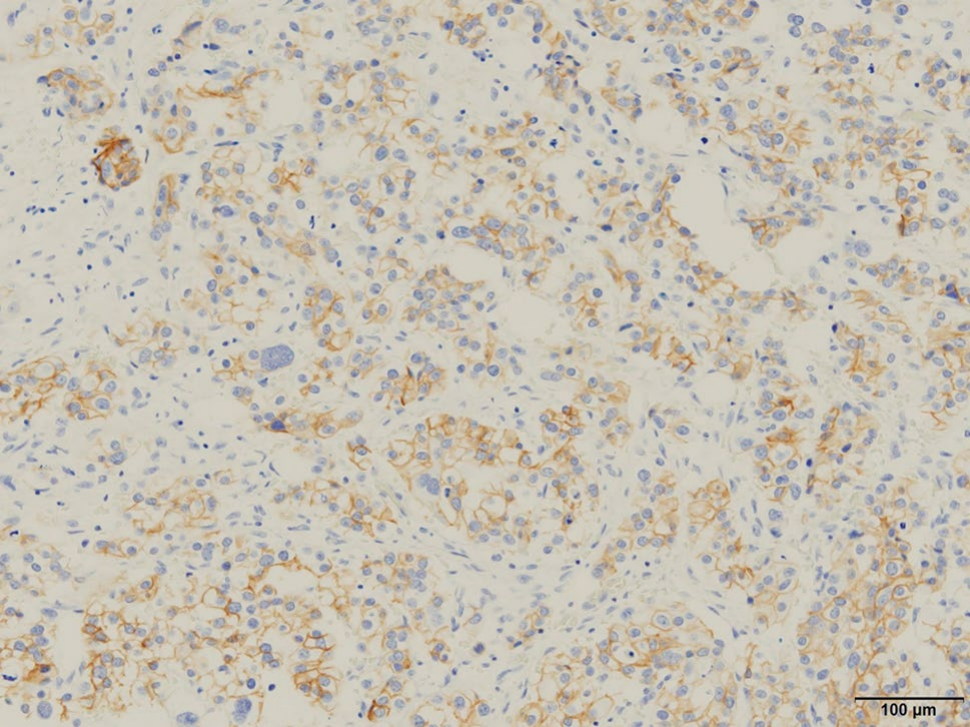
Positive HER2 expression in the metastatic lesion

Eight years after surgery (during adjuvant hormonal therapy), her serum CA15-3 level increased (230 U/mL), but she had no symptoms. FDG-PET/CT was then done, and slight FDG accumulation was found in the pelvic peritoneum (SUVmax: 3.7), and peritoneal dissemination of breast cancer was suspected (Fig. 1). Pelvic MRI showed no abnormalities. Upper and lower endoscopic examinations also showed no abnormalities. The presence of primary gynecological and gastrointestinal cancers was denied. Since there was no confirmation of peritoneal dissemination, diagnostic laparoscopy was performed. Peritoneal dissemination was observed in the omentum, the mesentery, and the peritoneum of the pelvis, and a mesenteric nodule was biopsied (Figs. 2, 3). Pathological examination showed peritoneal dissemination of breast cancer, but the biological markers were different from the primary lesion: ER(−), PgR(−), and Her2:3 + (Figs. 4, 5). Additional immunostaining was performed, which was positive for GATA-3 and negative for TTF1, and the diagnosis of metastatic breast cancer was confirmed. Breast screening was performed to detect metachronous primary breast cancer, but there were no abnormalities.

**Fig. 6:**
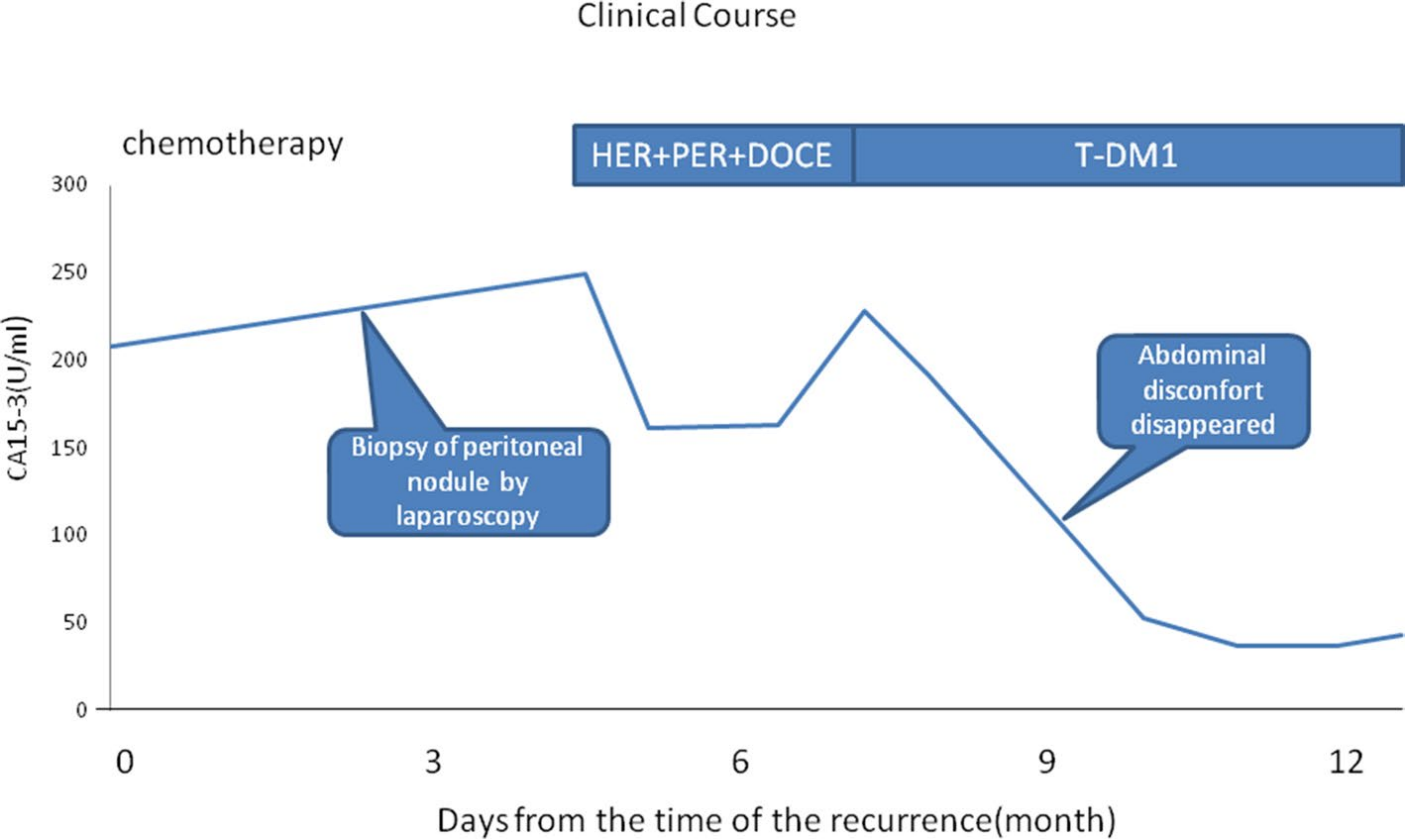
Clinical Course. Serum CA15-3 level decreased by T-DM1 therapy

Based on this result, perstuzumab, trastuzumab, and docetaxel combination therapy was started. Eight courses of perstuzumab therapy were completed, but peritoneal dissemination worsened, and she sometimes complained of stomach pain and diarrhea. Thus, the chemotherapy was changed to T-DM1. After several courses of T-DM1, her serum CA15-3 level decreased, and her systemic symptoms disappeared (Fig. 6).

## Discussion

Bertozzi et al. reported that peritoneal carcinomatosis of breast cancer had a prevalence of 0.7%. Peritoneal carcinomatosis is significantly more common with a high-grade, invasive lobular carcinoma and advanced TNM stage. Despite a high serum CA15-3 level, the metastasis site might not be identified by general diagnostic imaging [1]. Modern imaging techniques have low accuracy for the diagnosis of peritoneal carcinomatosis. CT scans have size limitations that are important for the detection of lesions, particularly in the small intestinal wall. FDG-PET/CT has high false-positive rates due to tissue inflammation after systemic therapy, as well as false-negatives due to metabolic inactivity of dormant neoplastic cells after chemotherapy [2]. Of course it is necessary to rule out other cancers, especially digestive tract and gynecologic cancers.

In such cases, laparoscopic examination should be performed. In gastric cancer and pancreatic cancer, it is always performed to determine whether radical resection is indicated [3–5]. In gastric cancer, staging laparoscopy is performed if the patient has type 3 or 4 or bulky lymph node metastasis and para-aortic lymph node metastasis [3]. In such cases, positive peritoneal dissemination or positive lavage cytology is found in 42.7–53.4% of cases [6–11]. On the other hand, complications such as intestinal injuries occur in 0–2.9% of cases [6, 7, 11–13]. In pancreatic cancer, staging laparoscopy can not only diagnose peritoneal dissemination but also determine the indication for resection when combined with intraoperative laparoscopic ultrasonography [4, 5]. Overall survival after the diagnosis of breast cancer metastasis was shorter in women affected by peritoneal metastasis than in women with other metastases [1]. Therefore, it is necessary to detect peritoneal dissemination earlier.

Biopsy of metastatic lesions is very important, particularly in the case of relapses during adjuvant hormonal therapy, because biomarker changes may occur. Increasing HER2 expression levels from 0, 1 +, or 2 + in the primary lesion to 3 + or FISH score > 2 in the metastatic lesion was observed in 10% of metastatic breast cancer cases [14]. HER2 expression is very important information for systemic treatment, and if a change in HER2 expression has occurred but is not detected, inappropriate therapy may be given.

In conclusion, the diagnosis of peritoneal carcinomatosis is often difficult. In such cases, laparoscopy can easily confirm peritoneal carcinomatosis. Since the biomarkers of metastatic lesions may sometimes change, laparoscopic biopsy is very useful and helps optimize the patient’s treatment.
